# Phytochemical Statistical Mapping of Red Grape Varieties Cultivated in Romanian Organic and Conventional Vineyards

**DOI:** 10.3390/plants12244179

**Published:** 2023-12-15

**Authors:** Cristina Mihaela Nicolescu, Marius Bumbac, Cristiana Radulescu, Claudia Lavinia Buruleanu, Radu Lucian Olteanu, Sorina Geanina Stanescu, Laura Monica Gorghiu, Bogdan Catalin Serban, Octavian Buiu

**Affiliations:** 1 Institute of Multidisciplinary Research for Science Technology, Valahia University of Targoviste, 13 Sinaia Alley, 130004 Targoviste, Romania; cristina.nicolescu@valahia.ro (C.M.N.); cristiana.radulescu@valahia.ro (C.R.); radu.olteanu@valahia.ro (R.L.O.); geanina.stanescu@valahia.ro (S.G.S.); 2 Faculty of Sciences and Arts, Valahia University of Targoviste, 13 Sinaia Alley, 130004 Targoviste, Romania; laura.gorghiu@valahia.ro; 3 Faculty of Environmental Engineering and Food Science, Valahia University of Targoviste, 13 Sinaia Alley, 130004 Targoviste, Romania; 4 Research Centre for Nanotechnologies and Carbon-Based Nanomaterials, National Institute for Research and Development in Microtechnologies—IMT Bucharest, 126 A Erou Iancu Nicolae Str., 077190 Voluntari, Romania; bogdan.serban@imt.ro (B.C.S.); octavian.buiu@imt.ro (O.B.)

**Keywords:** red grapes, organic/conventional vineyard, phenolics, flavonoids, multidimensional analysis

## Abstract

Red grapes are rich in phytochemicals such as phenolics and flavonoids, which are strongly correlated with their antioxidant activity. Thus, grapes as-harvested and grape extracts, especially those obtained from their seeds and pulp, have been reported to have health benefits, and accordingly, grapes and their derivatives are considered potential functional food ingredients. The total phenolic content, total flavonoid content, and the antioxidant activity of skin, pulp, and seeds of four grape varieties grown both in conventional and organic vineyards were examined in this study. Phytochemical characteristics of one native Romanian variety, Feteasca Neagra, were compared with data measured for three red grape varieties more commonly cultivated worldwide (Merlot, Pinot Noir, and Muscat Hamburg). It was found that the seeds of the Pinot Noir variety grown in an organic system contained the highest total phenolics of 169.53 ± 7.32 mg gallic acid equivalents/g and the highest total flavonoid content of 388.25 ± 10.72 mg quercetin equivalents/g, values corresponding to high antioxidant activity (312.84 ± 12.81 mg ascorbic acid equivalents/g). The total flavonoid content in the hydroalcoholic extracts obtained from seeds of Pinot Noir (organic vineyard) was around 24.5-fold higher than that of the skin of Pinot Noir (conventional vineyard). Experiments showed that seeds of all four tested grape varieties are good sources of total flavonoids, not only of total phenolics. When referring to the organic vineyard, the skin and pulp grapes showed good results for the total phenolic content. The antioxidant activities of the hydroalcoholic extracts were well-correlated with the total phenolic content and total flavonoid content. Lower values of these parameters were found for extracts obtained from skin and pulp than for those obtained from seeds of the same grape variety regardless of the culture management system (organic/conventional). Data mining techniques such as regression analysis, principal component analysis, and clustering analysis were applied to establish the potential correlation between the phytochemical content and the antioxidant activities of the red grapes on the one hand, and grape variety, anatomical parts, and vineyard type (organic/conventional) on the other hand.

## 1. Introduction

Along with other crops, vineyards are providing products with high nutritional value for human consumption. Fresh or processed grapes, together with other fruits and vegetables, have been associated with reduced risks of chronic diseases [1]. Many research studies in recent years have shown that phytochemicals, plant-derived molecules endowed with antioxidant effects, act as good fighters against chronic diseases. The antioxidant properties of plant foods are a result of the cumulative and synergistic activities of their bioactive molecules. The investigation of the role of vegetable food antioxidants in the prevention of many diseases requires a comprehensive database of plants used as raw materials [2,3,4].

The Vitis genus, with its several thousand varieties, is characterized by high levels of genetic diversity. The Vitis International Variety Catalogue identifies 21,045 names of varieties, including 12,250 for *V. vinifera*, but this includes a considerable number of synonyms and homonyms. The actual number of vine varieties for the *V. vinifera* species is estimated at 600,012 [5].

According to Eurostat, the European Union (EU) had 3.2 million hectares of vines in 2020, equivalent to about 45% of the world’s wine-growing areas. Out of these, Romania cultivated 183.7 thousand hectares, ranking the fifth member state at the EU level [6]. In Romania, grape production was 808.76 thousand tons in December 2022 and reached a record high of 1140.57 thousand tons in December of 2018 and a record low of 733.11 thousand tones in December 2016 [7].

It is worth mentioning that in the wine production industry, approximately 20–25% of the total processed grapes turn into a solid waste named grape pomace or grape marc, consisting of skins, residual pulp, seeds, and stems, and this also contains useful phytochemicals. Valorization of grape pomace biomass requires comprehensive studies towards optimization of the field, including the influence of the extraction methods, the culture management, or the vineyard type [8,9].

The consumer’s tendency to qualify organic cultivated fruits and vegetables as healthier has increased in recent decades, and consequently, these types of crops have been developed to align with market requests. Thus, the organic culture management type was applied in Romania on 2401 hectares in 2021, while in 2011, this area was only 842 hectares, which corresponds to an increase of 2.85 times in this 10-year timeframe. At the European Union level, the increase was significantly higher, namely 13.4 times, from 21,524 hectares in 2011 to 288,423 hectares in 2021 [10,11].

Food regulations, as indicated by the World Trade Organization (WTO) agreements and the European Union, aim to eradicate the line between conventional processed foods and functional foods by 2050, creating the premises to make nutraceuticals a significant part of the food market share in the EU [12,13]. In this respect, researchers need to focus on the identification of the mechanisms related to health benefits associated with natural/fresh foods (e.g., grapes) and derived products, and also on possible synergistic effects with other food compounds [14,15,16,17,18].

Significant interest is currently recorded by the scientific community for subjects related to the health effects of the phytochemicals from plant food. Potential health benefits of compounds like flavonoids (i.e., isoflavones) [14] and other polyphenolic compounds (i.e., resveratrol) in cardiovascular diseases, cancer, osteoporosis, cognitive decline, etc., are evaluated [19]. The protective effects and mechanisms of action of phytochemicals are discussed, and potential food safety issues are considered.

**Figure 1:**
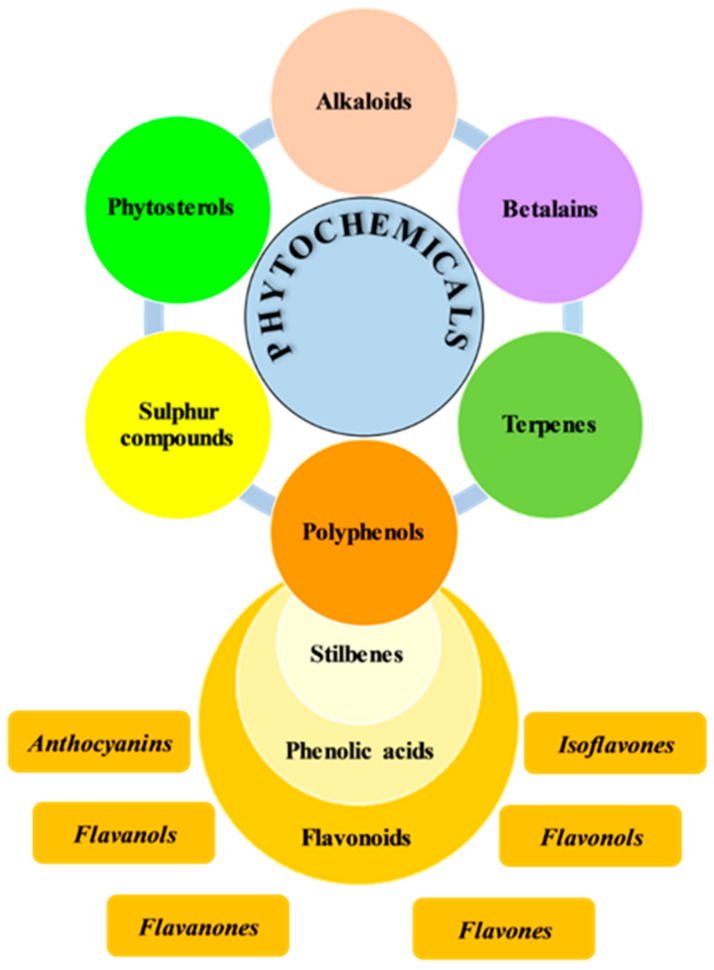
Classification of phytochemicals.

Phytochemicals are generally classified into polyphenols, terpenoids, alkaloids, phytosterols, and organosulfur compounds, as shown in Figure 1.

Flavonoids, a ubiquitous group of natural polyphenolic compounds, are one of the most common classes of compounds in fruits, vegetables, and plant-based beverages. Flavonoids are considered dietary supplements that promote health and prevent diseases [20]. The potential health benefits of red grape flavonoids refer to supporting the antioxidant cellular defense and proper functioning of the nervous system (anthocyanins—cyanidin, pelargonidin, delphinidin, malvidin), maintaining the health of the heart (flavanols—catechins, epicatechins, epigallocatechin) and maintaining the health of the urinary tract and heart (procyanidins and proanthocyanidins).

The constant interest in the biological activity of organically grown grapes and grape by-products contributes to their capitalization as a source of bioactive compounds with potential applications in the cosmetic, pharmaceutical, and food industries [21,22,23,24]. A full understanding of the phytochemical composition and antimicrobial activity of different anatomical parts of grapes of the species *Vitis vinifera* L., depending on the variety and the culture system, may support the development of new applications in these fields (i.e., nutraceuticals).

In this regard, a brief description of four grape varieties that were studied in this research work is given in Table 1, where the native Romanian variety Feteasca Neagra is compared with three other red grape varieties most commonly cultivated.

While grapes are primarily valued by consumers for their sensory attributes (sweetness, juiciness, and aroma), they serve as a significant source of health-promoting compounds [25]. The aroma of grapes, in particular, plays a vital role in their overall consumer appeal [26,27], and is related to monoterpenes [28]. These compounds were reported to have potential pharmacological properties, including antifungal, antibacterial, antioxidant, anticancer, and anti-spasmodic effects [29]. Some epidemiological studies [30,31] have even suggested that monoterpenes might have potential applications in the prevention and treatment of cancer types, such as breast, skin, lung, colon, and prostate carcinomas. Recent research reported on the anticarcinogenic and anti-mutagenic potential of tannins [32,33], showcasing their ability to combat a wide range of bacteria, yeasts, molds, and viruses [34].

Resveratrol (3,5,4’-trihydroxystilbene), a compound belonging to the stilben family, is commonly found in the skin of red grapes, and has been the subject of scientific investigation due to its potential benefits for aging, type 2 diabetes, obesity, and cardiovascular disease [35].

**Figure 2:**
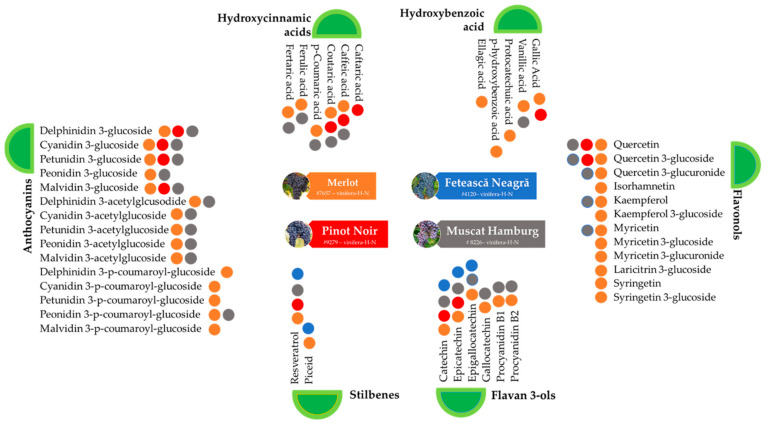
Phytochemical profiles of four red grape varieties—Feteasca Neagra compared with three well-known red grapes: Merlot, Pinot Noir, and Muscat Hamburg (blue, orange, red, and gray dots indicate presence of the specific bioactive compound in the respective red grape variety).

Figure 2 synthetizes available literature data on the phytochemical profiles of Feteasca Neagra, the native Romanian red grape variety studied, in comparison with the other three red grape varieties, most commonly cultivated worldwide. One may observe a scarcity of information for the Feteasca Neagra variety, though it is the most cultivated red grapevine in Romania, holding 7.26% of the total area for wines with PDO (protected designation of origin) [36].

**Table 1:** Characteristics of studied red grape varieties and their anatomical components.

Grape Variety	Brief Description	Anatomical Part		
		Skin	Seed	Pulp
Feteasca Neagra #4120—vinifera-H-N 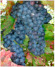	Grape bunches—14 cm long, cylindrical/conical-cylindrical, thick berries Grape berries—size of riped berry is 12–15 mm, must is colorless to slightly colored	thick blue-black plum colored, rich in tannins and sugar; violet reflexes	two seeds, hard outer shell, red-brown ~ 2–3% from the weight of berries ~ 1% of the total weight of grape contains 7–22% oil (dry basis) [37]	juicy, fruit taste accumulates 180–200 g/L sugars
Merlot #7657—vinifera-H-N 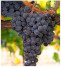	Grape bunches—cylindrical shape, 14–18 cm, average weight of 100–120 g Grape berries—dense, small (14 mm), spherical shape, black shade, juicy and slightly fragrant	thick, blue-black, fragrant rich in anthocyanins and sugars	two seeds, hard outer shell, red-brown ~ 2–4% from the weight of berries ~ 1–2% of the total weight of grape 8–20% oil (dry basis) [37]; polyphenols—catechin, epicatechin, trans-resveratrol, procyanidin B1 [38]	juicy taste accumulates 200–240 g/L sugars
Pinot Noir #9279—vinifera-H-N 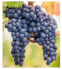	Grape bunches—cylindrical shape, length of 10–11 cm, average weight 90–100 g Grape berries—dense berries, even distribution on the stalks, spherical shape, crunchy, with size of 12–14 mm, black	thick blue-black, plum colored superior variety, with high concentrations of sugars and moderate acidity	two seeds, hard outer shell, red-brown ~ 2–4% from the weight of berries ~ 1–2% of the total weight of grape grape seed contains 9–20% oil (dry basis) [37]	juicy taste accumulates ~250 g/L sugars
Muscat Hamburg #8226—vinifera-H-N 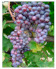	Grape bunches—branched with length of 18–25 cm, and weight of 325 g Grape berries—sparse, large, spherical to oval, fleshy; average mass/grain of 4.5 g; crunchy, aromatic taste	skin is thin, plum colored thick layer, black-bluish	one seed, hard outer shall, brown, or no seed [39]	crunchy, juicy, fine musky taste accumulates 160–220 g/L sugars

The applied treatment can influence the growth of plants, and also the chemical composition, in relation with the periodic biochemical treatments [40,41].

Mapping correlations between the type and levels of some bioactive compounds in grapes, and several independent factors such as cultivation system, origin of varieties, soil and climatic conditions, etc., may be used to optimize the culture yields and quality. The use of statistical analysis could provide important information to support the decision-making process for cultivars or other stakeholders in the field [42].

**Figure 3:**
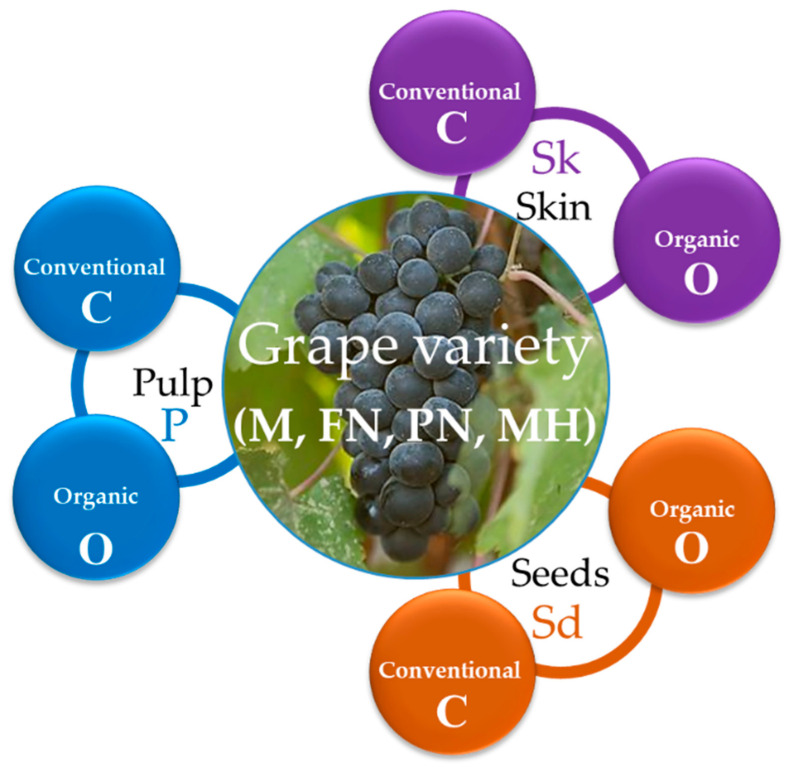
Acronyms in the manuscript for: (1) red grape varieties: M—Merlot, FN—Feteasca Neagra, PN—Pinot Noir, MH—Muscat Hamburg; (2) culture management: O—organic, C—conventional.

The study presented in this paper aimed at (1) applying in vitro non-invasive methods to extract bioactive compounds from grapes, (2) quantifying and comparing the levels of some extracted antioxidant species in three grape anatomical parts (skin, pulp, seeds), and (3) statistically mapping the bioactive compounds’ levels, considering the independent factors of management culture system (organic vs. conventional) and grape variety, for four grape varieties (Merlot, Pinot Noir, Feteasca Neagra, Muscat Hamburg) cultivated in Romania. For ease of reading, Figure 3 indicates the acronyms used throughout the manuscript.

## 2. Results

### 2.1. Physicochemical Characterization

#### 2.1.1. Infrared Spectroscopy Analysis

Antioxidants can be categorized into three groups based on their mechanisms of action: (i) primary antioxidants, which primarily act as scavengers to terminate free radicals, (ii) secondary antioxidants, serving as important preventive antioxidants by slowing down the initiation of chain reactions, and (iii) tertiary antioxidants, which focus on repairing damaged biomolecules [43,44].

Infrared spectroscopy may provide information on the structure of the compounds present in dry materials, and may thus help to obtain a general picture related to antioxidant species that may be extracted from the biomass into the solvent mixture.

**Figure 4:**
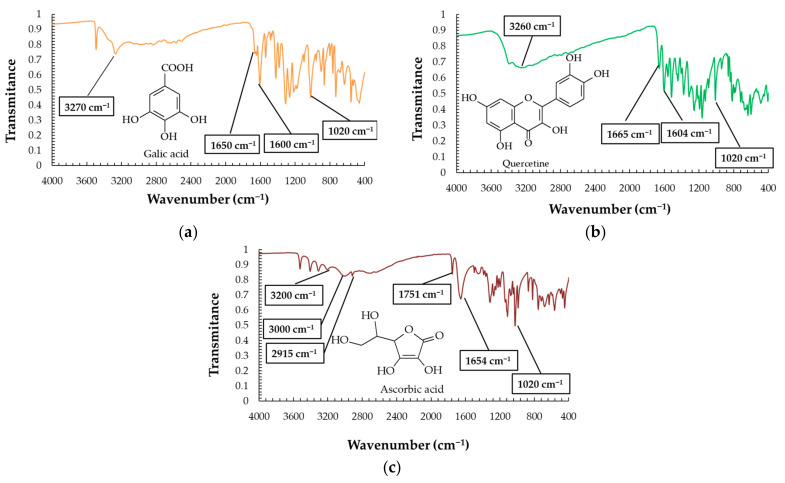
Infrared spectra of reference analytical-grade compounds (**a**) gallic acid, (**b**) quercetin, and (**c**) ascorbic acid, with indication of characteristic peaks, and chemical formulas.

**Figure 5:**
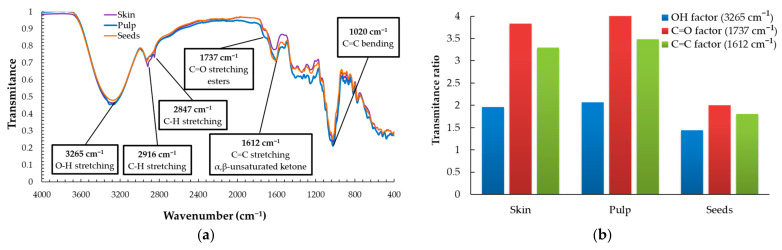
Infrared analysis of skin, pulp, and seeds of *Vitis vinifera* L.—Feteasca Neagra variety: (**a**) IR spectra of dry matter of grape fractions, and (**b**) functional group factor calculated relative to the 1020 cm^−1^ reference peak.

Figure 4 shows the infrared spectra of the three substances used as references in building the calibration curves in the ultraviolet–visible spectroscopic measurements applied in our study to quantify the total phenolic content, total flavonoid content, and antioxidant activity, respectively, as will be described further in the manuscript. One may consider that these spectra act like references for our study, as they show the absorption peaks of potentially present functional groups in the studied biomass of grape skin, pulp, or seeds. Figure 5 shows and compares the infrared spectra recorded for dried material of skin, pulp, and seeds of the Feteasca Neagra grape variety studied. Characteristic peaks identified, and their assignation, are provided by insets in Figure 5.

**Figure 6:**
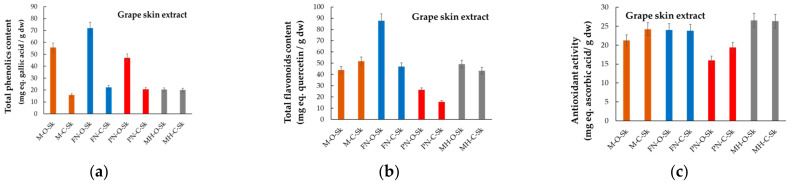
Comparative phytochemical profiles of grape skin extracts, (**a**) TPC, (**b**) TFC, and (**c**) AA, for grape varieties cultivated in Romania in organic and conventional management systems (acronyms as per Figure 3).

**Figure 7:**
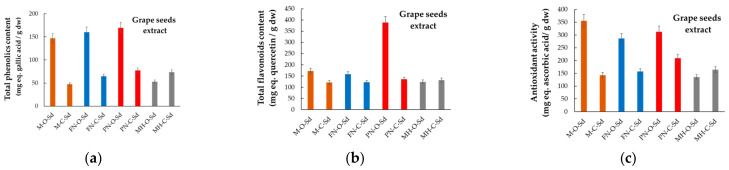
Comparative phytochemical profiles of grape seeds extracts, (**a**) TPC, (**b**) TFC, and (**c**) AA, for grape varieties cultivated in Romania in organic and conventional management systems (acronyms as per Figure 3).

**Figure 8:**
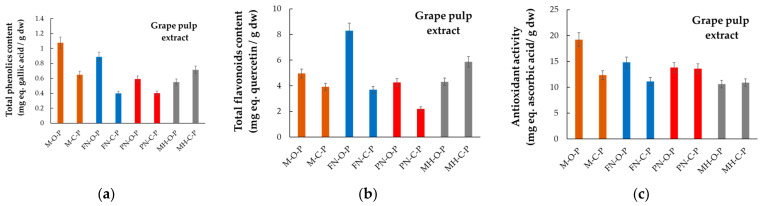
Comparative phytochemical profiles of grape pulp extracts, (**a**) TPC, (**b**) TFC, and (**c**) AA, for grape varieties cultivated in Romania in organic and conventional management systems (acronyms as per Figure 3).

The infrared spectra plotted in Figure 4 and Figure 5 highlight the presence of -OH, >C=O functional groups, and of the unsaturated α and β ketones. In all these spectra, one may observe the presence of the peak that is characteristic to the C=C bending vibration at 1020 cm^−1^. This peak was chosen as a reference peak to calculate the factor named transmittance ratio, to compare the characteristic vibrations for all the samples, as is shown in Equation (1) and Figure 5b. It may be noted that while in the skin and pulp of the Feteasca Neagra variety, the C=O factors have similar values, for the seeds, this factor shows values at almost half of them. Similar situations were found when comparing the transmittance ratios calculated for C=C stretching, and the values of skin and pulp were significantly higher than those of seeds. The variation in the C=O factor correlated with the C=C factor values may indicate the presence of higher quantities of α,β-unsaturated ketones in the skin and pulp than in seeds for the studied grapes. The OH factor showed no notable differences between the three anatomical parts of the red grape varieties studied. This finding may suggest that the concentration of species carrying OH groups is similar regardless of the anatomical part of the grape berry for all grape varieties, although the ratio between different classes of OH compounds is different. For example, the sugar content in the pulp is higher compared with seeds and skin, while the phenolic species are found in higher concentrations in seeds (Figure 6, Figure 7 and Figure 8). However, no anatomical part or grape variety stands out with an OH factor different from the average.

(1) $$Transmitance\;ratio=\frac{Transmitance}{Transmitance\;at\;1020\;{cm}^{-1}}$$

#### 2.1.2. Ultraviolet-Visible (UV-VIS) Spectroscopy Analysis

The total phenolic content (TPC), total flavonoid content (TFC), and antioxidant activity (AA), respectively, for each hydroalcoholic extract obtained from the four red grape varieties, harvested from both organic and conventional systems, are given in Figure 6, Figure 7 and Figure 8, in which the abbreviations of samples stand as per Figure 3. Data included are presented for grape anatomical parts (skin, seeds, and pulp). Measured values are expressed in milligrams of gallic acid (GA) equivalents (eq.) per g of dry weight (dw) sample for TPC, in mg of quercetin (Qt) equivalents per g of dry sample for TFC, and in mg ascorbic acid (AscA) equivalents per g of dry sample for the antioxidant activity. The literature provides similar data referring to TPC, TFC, and AA, but for grapes as a whole or pomace, and rarely for grape anatomical components [42,45,46,47]. Hardly ever do data refer to the chemical composition of the grapes from organic versus conventional vineyards.

According to data presented in Figure 6, Figure 7 and Figure 8, the experimental findings demonstrate the differences between the four red grape varieties, mainly in relation to the anatomical parts used to prepare the hydroalcoholic extracts, and also in relation to the vineyard management system (organic/conventional).

#### 2.1.3. Total Phenolic Content

The variation in phenolic substances is influenced by grape variety, genetic factors, agro-climatic conditions, ripening process, and extraction method [48]. In the case of extraction from a single grape variety, the phenolic composition depends on the anatomical portion (whole grape pulp, skin, or seeds). The grape extractable phenolic compounds represent 60–70% in seeds, 28–35% in skin, and only 10% or less in pulp [49].

The seeds of Pinot Noir had the highest total phenolic content (169.53 ± 7.32 mg GA-eq/g) among the analyzed samples, followed by Feteasca Neagra (159.92 ± 4.87 mg GA-eq/g), and Merlot (146.80 ± 6.53 mg GA-eq/g), respectively. All three samples came from organic vineyards. The skin of all four organic grape varieties provided average values of the total content of phenolics (Figure 6). However, three skin samples stood out with higher values of the phenolic content, and these were organic Merlot (55.69 ± 3.18 mg GA-eq/g), Pinot Noir (47.04 ± 1.87 mg GA-eq/g), and Feteasca Neagra (71.98 ± 4.04 mg GA-eq/g).

Regardless of the grape skin extracts analyzed, the total phenolic content was smaller than that reported for Tannat grape skin, characterized by a TPC value of 114.6 ± 10.5 mg GA-eq/g [50]. Using a different referential, a value of about 3000 mg GA-eq/100 g dry weight was reported for grape pomace extract of a Merlot variety [51], while Doshi et al. determined the total phenolic contents for a Merlot variety to be 41.7 ± 0.09 mg GA-eq/mL in seed extracts and 6.1 ± 0.04 mg GA-eq/mL in skin extracts [52].

In grape skin (*Vitis vinifera* L.)*,* phenolic compounds are established in the inner stratum of the cell wall [53], and the main free phenolic compounds found were caftaric acid (40.4 ± 3.6 mg/g), isoquercitrin (212.1 ± 12.8 mg/g), kaempferol (362.7 ± 45.0 mg/g), and resveratrol (149.2 ± 11.0 mg/g).

The studied seed extracts prepared from grape varieties grown in the conventional system also contained relatively high total phenolics ranging from 47.38 ± 0.90 mg GA-eq/g for M-C-Sd to 73.53 ± 1.37 mg GA-eq/g for MH-C-Sd. Far from these values, the group of extracts obtained from the skin of all four grape varieties was characterized by the smallest total phenolic content (i.e., only 15.82 ± 0.50 mg GA-eq/g for M-C-Sk).

Within the group of analyzed grape varieties, Muscat Hamburg was distinguished by having the smallest to average values of total phenolic content, although the pulp of the grapes grown in the conventional system (MH-C-P) had the highest total phenolic content (0.714 ± 0.05 mg GA-eq/g) among the three other grape varieties studied, which were grown in conventional vineyards (M-C-P, PN-C-P, and FN-C-P, respectively).

In conclusion, the seeds and pulp of the four organic varieties (Pinot Noir, Feteasca Neagra, Merlot, and Muscat Hamburg) are excellent providers of total phenolics, while the seeds and pulp of the four varieties coming from conventional vineyards may be also considered good sources of these phytochemicals. Also, our experimental findings suggest that the organic system of culturing had a positive influence on the amount of total phenolics accumulated in the anatomical parts of grapes, except for the Muscat Hamburg variety (seeds and pulp).

Analyzing the total phenolic contents of 14 wine grape varieties, Yang et al. (2009) reported that Pinot Noir had the highest total phenolic content at 396.8 ± 12.4 mg of gallic acid equivalents (GA-eq)/100 g of grape [54].

Grape seeds are a complex matrix containing valuable compounds [53], and the polyphenols, among the most studied, were reported to reach about 7% [55]. In grape seed oil, the amount of polyphenols is 59–360 mg GA-eq/kg [56]. The unfiltered oil is indicated to have a high quantity of polyphenols, due to their hydrophilic nature.

Our data are in agreement with those reported by Pastrana-Bonilla et al. (2003), who found the highest concentration of phenolic compounds in seeds (about 2178.8 mg GA-eq/g) [57]. Contrary to our result, the authors determined a total phenolic level of 374.6 mg GA-eq/g in the grape skin, while the grape pulp contained 23.8 mg GA-eq/g.

Determination of the total polyphenol content of grape seeds, a by-product of the wine industry, led, for the Pinot Noir variety, to a value of 334.38 mgGA-eq/g dry extract, lower that same parameter measured for Novac variety seeds of 394.57 mgGA-eq/g dry extract, while seeds of a Merlot variety contained a total polyphenol content of 230.45 mgGA-eq/g dry extract [58].

The phenolic content of the seeds can vary from 5–8% by weight. Grape seed extracts are mentioned as an excellent source of proanthocyanidins, usually oligomers and polymers of polyhydroxy-flavan-3-oils, such as catechin and epicatechin, many in the form of gallate or glycosides [49]. Grape seeds consisted also of a lower content of phenolic acids than grape skin [48].

Grape seed extracts rich in polyphenols have been used to reduce the formation of acrylamide during the Maillard reaction. In a model system, the grape skin extracts showed a higher activity in decreasing acrylamide formation compared to seed extracts, probably due to the combination of polyphenols and the Maillard reaction products, which block the formation of acrylamide [59]. The phenolic compounds contribute also to the nutritional value of grape seed oil, together with the unsaturated fatty acids [60].

Grape skin is rich in bioactive compounds such as dietary fiber, phenolic acids, anthocyanins, flavonols, resveratrol, and proanthocyanidins. Some in vivo studies have shown that bioactive skin grape compounds improve glutathione metabolism and reduce apoptosis [61]. The bioavailability of compounds from the grape skin extract of the Tannat variety has been demonstrated in vitro [50]. Thus, it was concluded that these compounds have the potential to modulate key biochemical activities involved in the pathogenesis of diabetes and the control of hyperglycemia caused by this disease.

The beneficial effects of grape polyphenols on glucose metabolism depend on the different classes of polyphenols and not on the individual fractions, as demonstrated in a medium-term (8 weeks) clinical study [62].

The phenolic compounds exert various other biological activities, such as DNA protection against oxidative stress via radical scavenging activity [63] and neuroprotection [64], and integrated procedures for their extraction and encapsulation are currently being widely developed.

#### 2.1.4. Total Flavonoid Content

Pinot Noir (organic vineyard seeds) had the highest total flavonoid content (388.25 ± 10.72 mg Qt-eq/g) among the analyzed samples, followed by Merlot (172.19 ± 9.67 mg Qt-eq/g determined in seeds of the organic variety) and Feteasca Neagra (158.36 ± 11.10 mg Qt-eq/g, seeds—organic system), respectively. Our data are in agreement with those reported by Yang et al. (2009), who stated that out of the 14 grape varieties studied [54], Pinot Noir had the highest total flavonoid content, at 301.8 ± 6.2 mg/100 mL.

The skin of the organic grape varieties had higher amounts of total flavonoids when compared to the pulp of the same varieties. This was similar if the groups (pulp and skin) of grapes grown in a conventional system were analyzed.

An overview of the data underlined that contrary to the situation reported for total phenolic content, the skin of the grape could be considered a good source of flavonoids, and the same consideration stands for the grape seeds. When comparing these two anatomical parts, a low content of total flavonoids was determined for the skin of Pinot Noir grown in both conventional (15.79 ± 1.51 mg Qt-eq/g for PN-C-Sk) and organic (26.28 ± 1.46 mg Qt-eq/g for PN-O-Sk) systems.

According to data from Figure 7b, grape seeds could be good flavonoid providers, regardless of the culture system and grape variety. The total flavonoid content of the hydroalcoholic extracts obtained from seeds was higher than 120 mg Qt-eq/g, though an exception was noted for the sample PN-O-Sd.

The total flavonoid contents in seed and skin extracts of two grape cultivars were reported as follows: 26.2 ± 0.7 mg/mL (seed) and 2.9 ± 0.04 mg/mL (skin) for Merlot variety, and 30.5 ± 0.7 mg/mL (seed) and 2.8 ± 0.11 mg/mL (skin) for Pusa Navarang variety [52]. These values, however, cannot be fully compared with our results, as the data were expressed in mg/mL extract, and also because the solvent used by the authors was an aqueous solution of 80% methanol (*v*/*v*).

#### 2.1.5. Antioxidant Activity

The seeds of grapes from organic agriculture were characterized by high values of antioxidant activity. Merlot had the highest antioxidant activity (355.77 ± 9.57 mg AscA-eq/g) among the analyzed samples, followed by Pinot Noir (312.84 ± 12.81 mg AscA-eq/g), Feteasca Neagra (286.58 ± 10.47 mg AscA-eq/g) and Muscat Hamburg (135.77 ± 8.14 mg AscA-eq/g), respectively. Our data are in agreement with those reported by Tița et al. (2021) for Pinot Noir seeds, where an average value of 241.07 mg AscA-eq/g dry extract was mentioned by these authors [58].

High antioxidant activities were determined for the hydroalcoholic extracts of the grape seeds grown in a conventional agricultural system. Except for Muscat Hamburg (MH-C-Sd), the highest values were registered for the corresponding organic grape varieties.

Regardless of the vineyard type, the lowest antioxidant activities were determined for the hydroalcoholic extracts obtained from skin and pulp of the four grape varieties. In both conventional and organic systems, the antioxidant activity of the skin was a slightly higher than the antioxidant activity of the pulp of the same grape variety. Comparatively, the antioxidant activity of Merlot (organic culture, seeds, M-O-Sd) showed a 18.5-fold difference when compared to the antioxidant activity of Merlot (organic culture, pulp, M-O-P). The antioxidant activity of Pinot Noir (conventional culture, seeds, PN-C-Sd) was around 10.8-fold higher than that of Pinot Noir (conventional culture, skin, PN-C-Sk).

Although high amount of total phenolics were determined for the extracts obtained from pulp of red grape varieties, it seems that these data were not correlated with the corresponding antioxidant activities. A similar situation can be reported for the skin of the organic grape varieties.

Yang et al. (2009) determined a total antioxidant activity of 122.4 ± 5.7 μmol of vitamin C equivalents/g fresh grape, the highest value corresponded to Cabernet Franc (149.0 ± 10.0 μmol vitamin C equivalents/g). The authors established a strong correlation between total antioxidant activity and total phenolics of the analyzed grapes [54].

Cell-wall-linked phenolic compounds and non-cell-wall-linked phenolic compounds have been reported in plants [53], and this in an important aspect when their biological activity is discussed. Physical characteristics of the cell wall and plant environment interactions influence the phenolics’ mechanisms of action, where an antioxidative function of the vacuolar phenolic compounds is exerted when the physical barrier is broken.

As the phenolic compounds are the most important secondary metabolites with antioxidant properties in grapes, the total content of phenolic compounds in grape pomace extracts is usually well-correlated with their antioxidant activity. For this reason, grape pomace extracts have been used as food protection factors for preventing lipid oxidation in fish products and for their antimicrobial activity against various bacterial strains [65].

Antioxidants derived from grapes, rich in polyphenols, show promising prospects for addressing oxidative stress. Therefore, it is crucial to assess the antioxidative attributes of phenolic compounds, with numerous methodologies detailed in the existing literature. However, it is important to note that the quantity of individual antioxidants in a particular food item does not always indicate its overall antioxidant capacity. Instead, one should consider the synergistic interactions between various molecules within the food matrix in this regard.

### 2.2. Statistical Data Analysis

The large number of data and independent variables of interest led to the need to identify and quantify the potential interactions between them.

**Figure 9:**
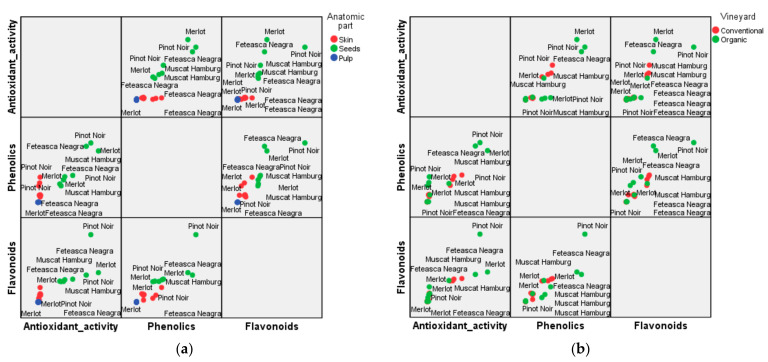
Correlations between the variables of interest (AA, TPC, and TFC), depending on the anatomical part of grapes (**a**) and vineyard type (**b**).

The matrix of the variables of interest (phytochemical content of the hydroalcoholic extracts of skin, pulp, and seeds, respectively, of the four red grape varieties and their antioxidant activity) is shown in Figure 9, with the markers being settled by anatomical part (a) and type of vineyard (b).

In giving an overview of the graphical representation, we emphasize that hydroalcoholic extracts obtained from grapes’ seeds had high amounts of phytochemical compounds and high values of antioxidant activity, regardless of the type of culture management and the grape variety, while the rest of the data overlap. Excepting the Muscat Hamburg variety, the parameters of interest showed higher values for hydroalcoholic extracts obtained from the seeds of the grapes grown in an organic system when compared to a conventional one. The extract from the seeds of the Pinot Noir variety (organic vineyard PN-O-Sd) contained higher amounts of phenolics and flavonoids and was characterized by a high antioxidant activity, very close to the antioxidant activity of the hydroalcoholic extract obtained from the seeds of the organic variety Merlot (M-O-Sd), which was the higher value within the set of the analyzed data.

Referring to the skin of the red grapes, Feteasca Neagra from organic vineyard (FN-O-Sk) was a good provider of phytochemicals, and it was also characterized by a high value of antioxidant activity. The Merlot variety cultivated in the conventional system (M-C-Sk) had a high amount of flavonoids (ranking the second in the group of studied extracts) and high antioxidant activity when compared to the Merlot variety cultivated in the organic system (M-O-Sk). In general, the hydroalcoholic extracts obtained from the skin of organic varieties could be differentiated through high values of the phytochemical content, with the above-mentioned exception. An average value of 19.36 ± 1.99 mg AscA-eq/mL was recorded for the antioxidant activity of the extract obtained from the skin of the conventional variety Pinot Noir (PN-C-Sk). This value was high comparing to the same parameter measured for the extract of the same variety grown in the organic system (PN-O-Sk).

When compared to conventional cultures, varieties Merlot, Feteasca Neagra, and Pinot Noir cultivated in the organic system were characterized by higher amounts of phytochemicals, and higher antioxidant values for the hydroalcoholic extracts from the grape pulp. Within the group of the experimental variants, the Muscat Hamburg variety grown in the conventional system (MH-C-P) was ranked in an intermediate position when comparing all the data referring to phytochemical compounds and antioxidant activity of the hydroalcoholic extracts obtained from the grape pulp.

### 2.3. Correlation between the Physicochemical Parameters and the Independent Factors Analyzed

Correlations between the amount of determined phytochemicals (total phenolics, total flavonoids, antioxidant activity) of grape seeds, skin, and pulp and the potential factors of influence were investigated. Because large amounts of data were obtained through chemical analysis, and given the low correlation between some pairs of parameters, a multivariate analysis was performed to unravel whether relationships existed between the variables of interest.

Analysis of categorical and continuous data combinations was performed using the *t*-test.

The null hypothesis in this case was that there is no significant difference between the variables of interest (TPC, TCF, and AA) of conventional and organic vineyards. All the four grape varieties and their anatomical parts were compared using a paired-samples *t*-test. If the test gave an output < 0.05, the null hypothesis was rejected. The *p*-values from the test are summarized in Table 2. It was observed that, regardless of the grape variety (Merlot, Feteasca Neagra, Pinot Noir, Muscat Hamburg) and anatomical part (skin, seeds, pulp), no significant differences were recorded between the conventional group and the organic one.

ANOVA was performed on the AA, TPC, and TFC of all the samples at a 5% level of significance. The results of the ANOVA are presented in Table 3. It turned out that there were no significant differences between AA, TPC, and TFC of the four grape varieties. Additionally, the AA, TPC, and TFC of grapes grown in the conventional vineyard were not statistically different from those determined for grapes grown in the organic vineyard. The ANOVA test highlighted that only the anatomical part had a significant effect on AA, TPC, and TFC.

According to the univariate analysis of variance, there were main effects but no interaction between the independent factors discussed (i.e., grape variety, type of vineyard, and anatomical parts).

**Figure 10:**
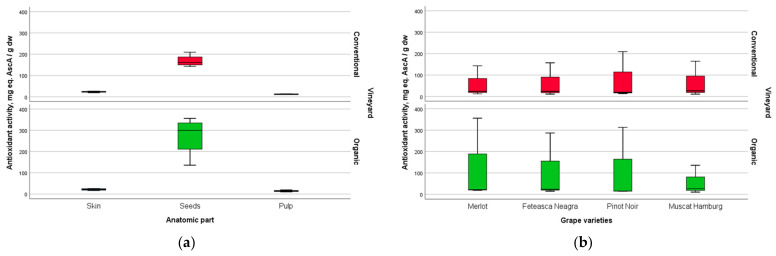

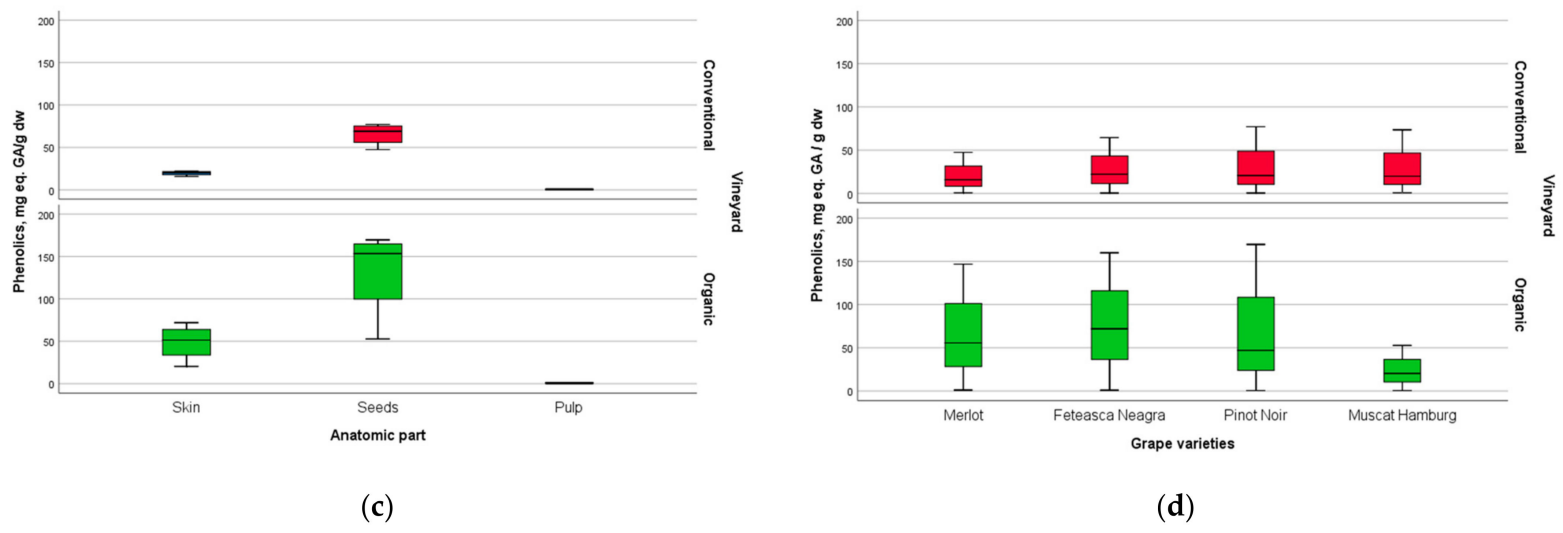
Boxplots of data in relationship with the vineyard type (conventional/red versus organic/green) for AA (based on: (**a**) anatomical part, and (**b**) grape variety) and TPC (based on: (**c**) anatomical part, and (**d**) grape variety).

The influence of an organic system versus a conventional one on AA and TPC values is underlined in Figure 10. The boxplots emphasize that extracts obtained from seeds of grapes sampled from the organic vineyard were characterized by a high antioxidant activity and high phenolic content compared with those obtained from the seeds of the grapes grown in a conventional system. If the grape variety was used as the factor for weighting the differences between the dependent variables in terms of the influence of the type of vineyard, it was obvious that except for Muscat Hamburg, the other three grape varieties (Merlot, Feteasca Neagra, and Pinot Noir) from the organic system contained significantly higher amounts of total phenolics than the corresponding grapes grown in the conventional system. The antioxidant activity followed the same trend, with the organic Merlot variety differentiable consistently from the conventional Merlot variety. It seemed that the conventional system of growing was more favourable for Muscat Hamburg, with the extracts obtained from this variety containing a high amount of phenolics and higher related values of antioxidant activity compared with the extracts from the Muscat Hamburg organic variety.

**Figure 11:**
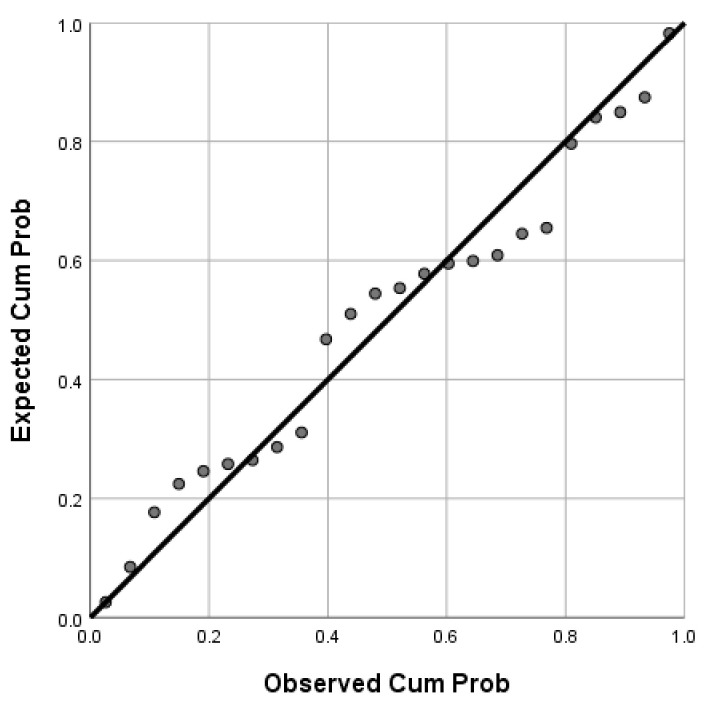
Normal probability plot of regression standardized residual (dependent variable: antioxidant activity)—black line represents the expected (estimated) values, while circles represent the determined values of the antioxidant activity.

The correlation between the independent variables (grape variety, growing system, and anatomical part of grapes) and dependent variables (AA, TPC, and TFC) was analyzed using multiple regression. The regression equation given below revealed the predicted antioxidant activity (Figure 11) of the 24 hydroalcoholic extracts for a high value of R squared (R^2^ = 0.915):

(2) $$Pred\left(AA\right)=-27.949+0.642\cdot G.V.-31.502\cdot Vineyard+30.812\cdot A.P.+1.911\cdot TPC+0.168\cdot TFC$$

where G.V. (grape variety) = 1/Merlot, 2/Feteasca Neagra, 3/Pinot Noir, and 4/Muscat Hamburg; Vineyard (vineyard type) = 1—Conventional, 2—Organic, and A.P. (anatomical part) = 1/skin, 2/seeds, and 3/pulp.

If only the antioxidant activity of extracts was predicted in the relationship with TPC and TFC, a high value of R squared (R^2^ = 0.853) was obtained, with the regression equation being the following:

(3) $$Pred\left(AA\right)=11.93+0.38\cdot TPC+0.224\cdot TFC$$

The degree of linear association between two quantitative variables following a bivariate normal distribution is described by the Pearson correlation. If the probability is lower than the significance level, the correlation coefficient is statistically significant.

Statistically significant values of the Pearson correlation coefficient (r) between the phytochemical compounds with antioxidant activity were recorded, as is shown in Table 4. For the other pairs of data, the bivariate relationships were not significant.

Spearman’s rank correlation test was applied to analyze the association (not necessarily linear) between the independent variables (anatomical part of grapes, type of vineyard, and grape varieties) and the dependent ones (TPC, TFC, and AA). Only the associations between the anatomical part of grapes and their bioactive compounds’ content was statistically significant (Table 5).

Factor analysis is used to identify variables or factors that can explain the model of correlation within the set of the observed variables. In this case, the observed variables were represented by the values of the antioxidant activity, total phenolic content, and total flavonoid content of the hydroalcoholic extracts obtained from the anatomical parts (skin, seeds, and pulp) of the four red grape varieties cultivated both in the conventional and organic system. Factor analysis is frequently applied to reduce the number of data and to identify a small number of factors that can explain the observed variance, but may also be used to generate a hypothesis referring to the mechanism of causality, or to analyze some aspects within variables before applying other statistical methods. Here, factor analysis was applied on the experimental data to identify potential factors generating the values of phytochemical characteristics for the hydroalcoholic extracts.

The extraction method used within the factor analysis was principal component analysis (PCA).

**Figure 12:**
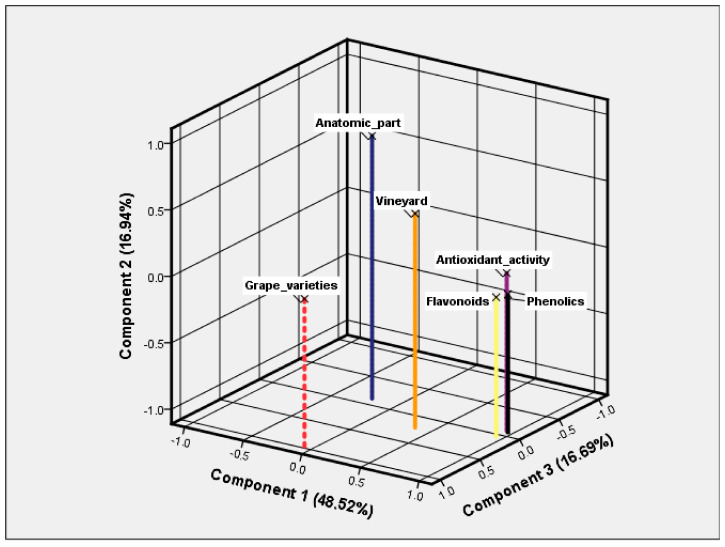
Component plot in rotated space (Varimax rotation).

The rotated matrix showed that the antioxidant activity, phenolic content, and flavonoid content formed the first factor (PC1), the anatomical part formed the second factor (PC2), and the grape varieties formed the third factor (PC3). These factors (Figure 12) accounted for a cumulative variance of 82.16% of the total variability in the dataset.

For each factor, relevant factor loadings higher than 0.6 were considered (Table 6).

In interpreting the first factor loadings, we emphasizes that the organic management system has a major influence on the increase in the content of total flavonoids and total phenolics, which determines the high values of antioxidant activity in the hydroalcoholic extracts. However, the vineyard type has a loading value lower than 0.6, and this shows that the phytochemical content of red grapes is also influenced by other factors than the culture system itself.

The grape anatomical part and the grape variety showed high factor loadings, so our factor analysis allowed the comparison of the phytochemical content and antioxidant activity of hydroalcoholic extracts, with estimates based on those two parameters suggesting that grape seeds and the Pinot Noir variety may provide important amounts of phenolics and flavonoids that can be taken into account for different practical applications (i.e., food industry, cosmetics, pharmaceutics).

**Figure 13:**
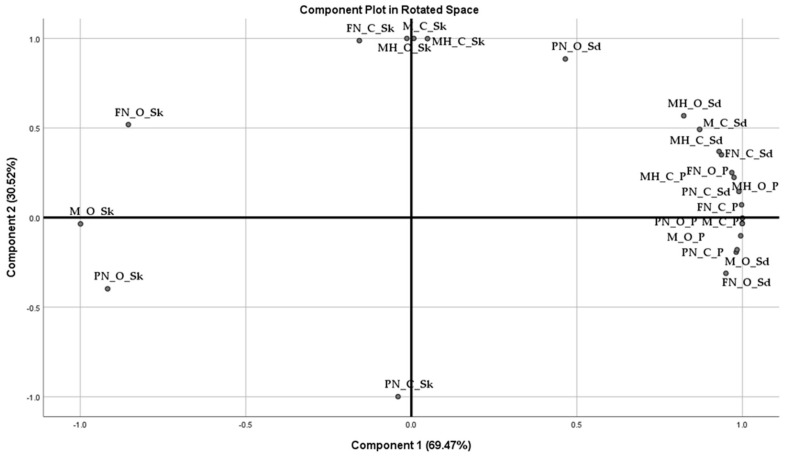
Component plot in rotated space (Varimax rotation) of the 24 hydroalcoholic extracts.

PCA is used widely in numerous fields, such as chemometrics and pomological studies [66]. The 24 extracts were separated via the first two components, accounting for 99.99% (Figure 13). The hydroalcoholic extracts were grouped, with the majority in the positive part of the first principal component, while in its negative dial remained, distinctly separated, the extracts obtained from the skin of Merlot, Feteasca Neagra, and Pinot Noir (only organic varieties). The positive part of the second principal component, explaining 30.52% of the total variance, suggested similarities between the TPC, TFC, and AA of extracts obtained from the skin of Muscat Hamburg (both conventional and organic varieties) and Feteasca Neagra (conventional variety) and the seeds of Pinot Noir (organic variety) and Merlot (conventional variety).

Cluster analysis was applied both on the experimental data (content of phytochemicals and antioxidant activity measured for hydroalcoholic extracts of the studied red grapes) and on the values of descriptors (grape variety, type of vineyard management, anatomical part of grapes). The scope of applying cluster analysis was to identify which hydroalcoholic extracts obtained from the skin, seeds, and pulp of the studied grape varieties from conventional and organic vineyards were similar to each other but at the same time different from the extracts from the other groups in terms of the phytochemical attributes (total phenolic content, total flavonoid content, and antioxidant activity).

Hierarchical cluster analysis allows for grouping the investigated compounds into homogeneous groups based on their common characteristics. This analysis is intended to find out if in the set of the investigated hydroalcoholic extracts, there are identifiable groups with similar characteristics (antioxidant activity, content of phenolics, and flavonoid compounds). Additionally, the analysis seeks to establish if the groups of studied hydroalcoholic extracts may be split depending on grape variety, type of vineyard, and anatomical part.

The method used in clustering was the Ward method on variables of the type interval, achieved by applying the squared Euclidean distance. The Ward linkage method analyzes the variances to assess the distances between clusters. The method is generally known to be effective, and cluster membership is assessed by calculating the total sum of the squares of the deviations from the average of the respective cluster. The criterion for merging clusters is to produce an increase in the sum of the squares of the errors that is as small as possible.

**Figure 14:**
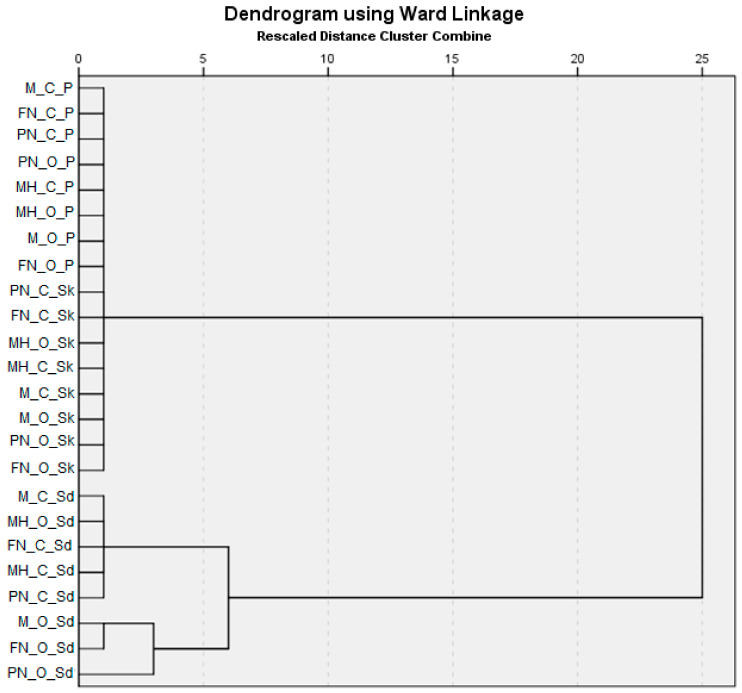
Dendogram of variables of interest (using Ward linkage).

The hierarchical cluster analysis proved to be statistically significant at a significance threshold of 5%. The dendrogram associated with the analysis is shown in Figure 14. The affiliation of each extract from red grapes to a cluster was obtained as follows: *Cluster 1*: M-C-P, FN-C-P, PN-C-P, PN-O-P, MH-C-P, MH-O-P, M-O-P, FN-O-P, PN-C-Sk, FN-C-Sk, MH-O-Sk, MH-C-Sk, M-C-Sk, M-O-Sk, PN-O-Sk, FN-O-Sk (16 extracts), *Cluster 2:* M-C-Sd, MH-O-Sd, FN-C-Sd, MH-C-Sd, PN-C-Sd (5 extracts), *Cluster 3:* M-O-Sd, FN-O-Sd (2 extracts).

One extract, belonging to the Pinot Noir variety (PN-O-Sd), remained isolated till the second stage of clusterization, when it aggregated to cluster 3. Further, both clusters 2 and 3 aggregated in the third stage of clusterization, including all the extracts obtained from seeds, regardless of grape variety and system of cultivation. The hydroalcoholic extracts obtained from skin and pulp of red grape varieties aggregated in the same cluster (cluster 1) in the first stage of clusterization.

The clusters extracted from our results confirmed important variations among the 24 hydroalcoholic extracts obtained from four varieties, three types of anatomical part, and two growing systems, in terms of antioxidant activity and TPC and TFC values.

Here, distance (X axis) is the Euclidean distance among hydroalcoholic extracts based on the investigated parameters (AA, TPC, and TFC). Two clusters formed initially, with the least similarity (smaller Euclidean distance, by 1) when compared to the remaining origins. The higher Euclidean distance (by 6) was found for the cluster containing all the extracts obtained from seeds. However, this value was small when compared with the results obtained for the others. In the literature, cluster analysis performed on mean values (physical traits, proximate composition, and antioxidant capacity) in *Z. lotus* fruit samples collected from eleven Moroccan origins revealed a smaller Euclidean distance of less than 10 [67].

The analysis of classification of the hydroalcoholic extracts based on their phytochemical content, antioxidant activity, and three descriptors (grape varieties, type of vineyard, and anatomical part of grapes) as variables revealed an optimum number of two classes. These clearly demarcated the anatomical parts of grapes, with seeds on the one hand and skin and pulp on the other hand.

## 3. Materials and Methods

### 3.1. Plant Material and Sample Preparation 

The grapes selected for this study were grown in the country of Romania, in Prahova county (conventional culture) and in Buzau county (organic system), in a temperate continental climate, with respective values for average temperature of 11.1 °C and 11.9 °C, for annual rainfall of 733 mm and 668 mm, and for altitudes of 165 m and 152 m. The collected grape samples belonged to a single cycle of grape growth and ripening.

The samples’ representability was insured as follows: grape clusters were manually harvested at the full maturity stage, from vines placed in similar positions in rows, and one cluster was collected from 100 vines of each grape variety using pruning scissors; fresh samples were brought to the laboratory within four hours; then, the berries were separated from the cluster in three parts corresponding to the studied anatomical parts, i.e., the skin, seeds, and pulp; each of these fractions contained mixed aliquots separated from each grape variety (i.e., all the grape skins of Feteasca Neagra variety grown in organic culture were manually mixed before next processing steps); for preservation reasons, skins and seeds were dried at 40 °C for 48 h, while pulps were frozen and maintained at −18 °C; the necessary aliquots were defrosted on the day of the lab tests; polypropylene containers were used for the storage of pulp samples, while the dried skins and seeds were kept in paper envelopes in a dry environment; the dried skins and seeds were ground and sieved to less than 2.5 mm; the quartering method was then applied to weigh the quantities needed to prepare the hydroalcoholic extracts; and three replicates of weighted aliquots were sampled for each studied grape variety and anatomical part, respectively.

Hydroalcoholic (50% *v*/*v*) extracts were prepared via classical maceration at room temperature, with magnetic stirring for 3 h, followed by maintenance in the dark for 21 h. The ratio of grape fraction mass to solvent volume was 1 to 25 g/mL for skin and seeds, while for pulp, this ratio was 6 to 25 g/mL.

Analytical-grade reagents were procured for all the experiments, while the deionized water used to prepare solutions, and as the washing solvent, was produced in-house at a quality of 0.056 µS/cm.

### 3.2. Phytochemical Measurements 

The phytochemical measurements were performed immediately after the total contact time with the solvent, i.e., after 24 h.

For the ultraviolet–visible spectroscopic investigations, the BioEvolution 201 spectrophotometer from Thermo Fischer Scientific (Waltham, MA, USA) was used, and the necessary wavelength was selected according to the lab protocol applied, as will be indicated below. Triplicate samples were used, and standard deviation was calculated for results reporting.

The infrared spectroscopy was performed on the Fourier Transform Infrared Spectrometer Vertex 80v (Bruker Corporation, Billerica, MA, USA), the sample positioning and scan system was attenuated reflectance (ATR), dried samples were scanned in the range of 4000–400 cm^−1^, and the average spectrum of 32 scans (with baseline and atmospheric correction) was declared as the result.

#### 3.2.1. Total Phenolic Content (TPC)

The Folin–Ciocalteu procedure was applied for this test, as previously described [68]. Absorbance measurements of a monochromatic radiation of 765 nm were recorded for samples placed in glass cuvettes with a 1 cm optical path. Gallic acid (GA) was used as the reference for the calibration, and results were expressed in mg GA-equiv./g (d.w.). The calibration curve was built in the range of 0.01–0.08 mg/mL gallic acid.

Analytical-grade reagents used for this assay were: Folin–Ciocalteu (FC) solution 2M, and sodium carbonate powder (purity > 99.5%), both procured from Sigma-Aldrich (St. Louis, MO, USA). Using these, solutions of Na_2_CO_3_ 8% (*w*/*w*), Folin–Ciocalteu 10% (*v*/*v*), and gallic acid dilutions for calibration (as mentioned above) were prepared.

The sample preparation procedure for the spectrophotometric readings consisted of the following algorithm: an aliquot of 0.5 mL grape extract was pipetted in a 5 mL Eppendorf tube; then, 2.5 diluted 10% FC reagent was added and mixed for 8 min; then, 2 mL of sodium carbonate 8% aqueous solution was added, mixed, and left at rest for 60 min before measurement. The same procedure was applied to prepare blank samples, while the volume of extract was replaced with deionized water.

#### 3.2.2. Total Flavonoid Content (TFC)

For this phytochemical parameter, the procedure based on the formation of Al^3+^–flavonoid colored complexes was applied, as described elsewhere [69], and the absorbances were measured at 510 nm, using 1 cm glass cuvettes. Calculations were performed using a calibration curve made with quercetin (Qt) at different dilutions (linearity in the range of 0.1–1.0 mg Qt/mL), and final results for grape extracts were expressed in mg Qt-eq./g (d.w.).

Analytical-grade reagents used for this assay were sodium nitrite (99% purity), aluminium chloride (99% purity), sodium hydroxide (purity > 97%, with sodium carbonate < 1%) from Merck Millipore, ethanol, and quercetin hydrate with 95% purity from Sigma-Aldrich. Using these, solutions of NaNO_2_ 5% (*w*/*w*), AlCl_3_ 10%, NaOH 1 M, and quercetin dilutions for calibration were prepared.

To prepare samples for the spectrophotometric readings, an aliquot of 1 mL extract was pipetted into a graduated flask of 10 mL, diluted with 4 mL deionized water, and then 0.3 mL of 5% sodium nitrite solution was added, mixed, and left at rest for 5 min; then, 0.3 mL of 10% aluminum chloride solution was added, mixed, and left at rest for 6 min; then, 2 mL of sodium hydroxide solution 1M was added, and the flask was filled to the mark with deionized water. The same procedure was applied to prepare blank samples, in which the 1 mL extract was replaced by 1 mL deionized water.

#### 3.2.3. Antioxidant Activity (AA)

The total antioxidant activity of the grape fractions was determined using the phosphomolybdenum complex formation method developed by Prieto et al., slightly modified according to the experimental conditions, as described previously [70,71], in a way considered more suitable when red grape extracts are investigated, as it avoids color interferences with the natural colors of the extracts.

The colored samples’ absorbances were measured at 700 nm, using 1 cm glass cuvettes. Calculations were performed using a calibration curve made with ascorbic acid (AscA) in different dilutions (linear range 0.0–300.0 mg AscA/L), and the final results for grape extracts were expressed in mg AscA-eq./g (d.w.).

Analytical-grade reagents used to prepare the Prieto reagent were trisodium phosphate dodecahydrate, ammonium molybdate tetrahydrate, sulphuric acid conc. 95%, and dimethyl sulfoxide. All these reagents, together with the ascorbic acid used to obtain the calibration curve, were procured by Sigma-Aldrich.

Colored samples were prepared in thermoresistant glass tubes by adding 200 microliters of grape extract, diluting with 9.8 mL deionized water and 5 mL Prieto reagent, mixing, and then incubating for 90 min at 95 °C, before allowing to cool at room temperature before the spectrophotometric readings. The same procedure was applied to prepare blank samples, and deionized water was added instead of grape extract.

### 3.3. Statistical Analysis

The phytochemical compounds of interest (phenolics and flavonoids) and the antioxidant activity of the hydroalcoholic extracts obtained from all the four red grape varieties—both from conventional and organic vineyards, and from the different anatomical parts (skin, seeds, and pulp) of the grapes—were taken into account through analysis using data mining techniques.

Data analysis was conducted using IBM SPSS Statistics 24.0 for MS Windows 10 (SPSS Inc., Chicago, IL, USA). The analysis of variance was performed to establish differences between grape varieties and their anatomical parts in terms of variables of interest.

Data were reported as the mean ± SD of three replications.

## 4. Conclusions

A growing awareness of the relationship between food and health is being observed among consumers worldwide. In this context, foods that promote and contribute to maintaining people’s health and well-being are well-represented in the fast-moving consumer goods sector. Grape pomace, consisting of the leftover after grapes are pressed for juice or wine, contains valuable components that can be further processed to extract useful products.

Grape seeds are proven to be important sources of total phenolics and total flavonoids and to have a high antioxidant activity, regardless of whether grape varieties are grown in an organic or conventional system. However, except for the Muscat Hamburg variety, higher amounts were determined for grapes coming from organic agriculture. The seeds of Pinot Noir (organic vineyard) were rich in total phenolics and total flavonoids, while the seeds of Merlot (organic vineyard) had the highest antioxidant activity. The results emphasize that the quantity of individual antioxidants in a specific food item may not necessarily reflect its overall antioxidant capacity. Instead, considering the synergistic interactions between different molecules within the food matrix may be effective.

The skin of conventional grape varieties was low in total phenolics. Generally, the grape skin and pulp were characterized by lower and the lowest values of antioxidant activity, respectively, regardless of the vineyard type.

The FTIR analysis of the dry skin, pulp, and seeds correlated with the TPC, TFC, and AA results of the hydroalcoholic extracts, which may indicate the presence of higher quantities of α,β-unsaturated ketones in skin and pulp than in seeds for the studied grapes.

Our comparative study of the hydroalcoholic extracts of the native Romanian grape variety Feteasca Neagra with the other three red grape varieties, Merlot, Pinot Noir, and Muscat Hamburg, showed valuable similarities in terms of antioxidant activity, as measured through the Prieto method and expressed in equivalents of ascorbic acid per dry weight unit. Additionally, it is worth mentioning that extracts obtained from skins of the Feteasca Neagra variety had higher values for both total phenolic content and total flavonoid content. On the other hand, it was observed that for the Feteasca Neagra variety, the vineyard type significantly influenced the phytochemical parameters of the extracts.

The data mining techniques applied to the set of experimental data consisting of the total flavonoid content, total phenolic content, and antioxidant activity of the hydroalcoholic extracts obtained from four red grape varieties indicated that the vineyard type (organic/conventional) has an influence on the values of these parameters, but the grape anatomical part and the grape variety should also be regarded as marker elements of the phytochemical properties of grapes.

## Figures and Tables

**Table 2:** *p*-values of paired *t*-test (*p* = 0.05).

Pairs	*p*-Values
M_C_Sk–M_O_Sk	0.590
M_C_Sd–M_O_Sd	0.127
M_C_P–M_O_P	0.309
FN_C_Sk–FN_O_Sk	0.186
FN_C_Sd–FN_O_Sd	0.086
FN_C_P–FN_O_P	0.144
PN_C_Sk–PN_O_Sk	0.324
PN_C_Sd–PN_O_Sd	0.102
PN_C_P–PN_O_P	0.313
MH_C_Sk–MH_O_Sk	0.356
MH_C_Sd–MH_O_Sd	0.085
MH_C_P–MH_O_P	0.282

**Table 3:** *p*-values of one-way ANOVA.

	Vineyard	Grape Variety	Anatomical Part
Antioxidant activity	0.441	0.948	0.000
Total phenolic content	0.131	0.837	0.000
Total flavonoid content	0.379	0.917	0.000

**Table 4:** Pearson correlation coefficient values and types of association for the analyzed variables.

Association	Pearson Coefficient/Type of Association	Type of Association	Level of Significance
Antioxidant activity * Total phenolics	0.916/strong	direct proportional	*p* = 0.01
Antioxidant activity * Total flavonoids	0.867/strong	direct proportional	*p* = 0.01
Total phenolics * Total flavonoids	0.888/strong	direct proportional	*p* = 0.01

* Cumulative effect of the analyzed variables

**Table 5:** Non-parametric test for association between dependent and independent variables.

Association	Spearman’s Coefficient	Type of Association	Level of Significance
Antioxidant activity * Anatomical part of grapes	0.457	indirect	*p* = 0.05
Total phenolics * Anatomical part of grapes	0.509	indirect	*p* = 0.05
Total flavonoids * Anatomical part of grapes	0.472	indirect	*p* = 0.05

* Cumulative effect of the analyzed variables.

**Table 6:** Factor loadings (Varimax normalized) using principal component extraction.

Factor	Eigenvalue	Cumulative Variance (%)	Antioxidant Activity	Phenolics	Flavonoids	Grape Varieties	Vineyard Type	Anatomical Part
Factor 1	2.91	48.45	**0.937**	**0.980**	**0.940**	−0.071	0.359	−0.229
Factor 2	1.01	65.32	0.078	−0.058	−0.062	0.000	**0.499**	**0.866**
Factor 3	1.00	82.16	−0.094	−0.045	0.044	**0.970**	0.208	−0.118

Bold values emphasize the higher values of the factor loadings related to each of the free factors (factor loadings greater than 0.6)

## Data Availability

Data are contained within the article.
